# 2-Brain Regulation for Improved Neuroprotection during Early Development (2-BRAINED): a translational hyperscanning research project

**DOI:** 10.3389/fpsyg.2024.1516616

**Published:** 2025-01-28

**Authors:** Lucia Billeci, Valentina Riva, Elena Capelli, Serena Grumi, Miriam Paola Pili, Maddalena Cassa, Eleonora Siri, Elisa Roberti, Renato Borgatti, Livio Provenzi

**Affiliations:** ^1^Clinical Physiology Institute, National Research Council of Italy (IFC-CNR), Pisa, Italy; ^2^Child Psychopathology Unit, Scientific Institute IRCCS E. Medea, Bosisio Parini, Italy; ^3^Department of Brain and Behavioral Sciences, University of Pavia, Pavia, Italy; ^4^Developmental Psychobiology Lab, IRCCS Mondino Foundation, Pavia, Italy; ^5^Child Neurology and Psychiatry Unit, IRCCS Mondino Foundation, Pavia, Italy

**Keywords:** EEG, hyperscanning, infant, parent, preterm, synchrony, video-feedback

## Abstract

**Introduction:**

Very preterm (VPT) birth is a major risk condition for child development and parental wellbeing, mainly due to multiple sources of stress (e.g., separation and pain exposure) during the neonatal intensive care unit (NICU) stay. Early video-feedback (VF) interventions proved effective in promoting VOT infants’ development and parental wellbeing. Electroencephalography (EEG) hyperscanning allows the assessment of brain-to-brain co-regulation during live interaction between infants and parents, offering promising insights into the mechanisms behind the interactive benefits of early VF interventions.

**Goals:**

This study aimed to compare indices of brain-to-brain co-regulation between dyads of full-term (FT) and VPT infants interacting with their mothers and investigate the effect of an early post-discharge VF intervention on the brain-to-brain co-regulation indices of VPT dyads.

**Methods and analysis:**

VPT and FT dyads will be enrolled at birth, and the former will be randomly allocated to one of two arms: VF intervention or care as usual. Short-term effectiveness will be assessed through ratings of mother–infant interaction videotaped before and after the VF intervention or care as usual. Mothers of VPT and FT infants will report on their mental state, parenting stress and bonding, and infant temperament and sensory profile at 3 and 6 months (corrected age, CA). At 9 months CA, all dyads will participate in a lab-based EEG-hyperscanning paradigm to assess brain-to-brain co-regulation through phase-locking value (PLV) and other explorative indices.

**Ethics and dissemination:**

This study was funded by the Italian Ministry of Health and received approval by the Ethics Committee of Pavia (Italy) and participating hospitals. Research findings will be reported in scientific publications, presented at international conferences, and disseminated to the general public.

**Study registration number:**

GR-2021-12375213 (Italian Ministry of Health registry).

## Introduction

Very preterm (VPT) birth is a major challenge for healthcare systems worldwide (Beam et al., 2020), representing the leading cause of long-lasting chronic diseases in childhood and child mortality (Ohuma et al., 2023). While VPT infants are exposed to heightened medical risk and several stress sources from the neonatal intensive care unit (NICU) environment (Cong et al., 2017; Provenzi et al., 2018), their parents also may face critical levels of psychological distress leading to increased risk for depression, anxiety, and parenting stress in the postpartum period (Caporali et al., 2020).

Notably, biobehavioral dysregulation patterns have been observed in dyads of VPT infants and their caregivers in the first months of life (Jean and Stack, 2012; Montirosso et al., 2010; Neugebauer et al., 2022; Provenzi et al., 2019), suggesting that VPT birth and NICU-related stress may affect child development and parental adjustment by altering critical processes of dyadic co-regulation during the first 1,000 days (Feldman, 2006; Linnér and Almgren, 2020). Early interventions that promote parent–infant closeness may help foster the establishment of similar psychobiological co-regulatory processes (Ionio et al., 2021; Lordier et al., 2019; Mörelius et al., 2015; Welch and Ludwig, 2017), providing buffering and protective benefits for both child development and parental wellbeing (Burke, 2018; He et al., 2021; Thomson et al., 2020).

Video-feedback (VF) interventions are well-validated parenting support programs that focus on parent–infant closeness, promote parental sensitivity, facilitate co-regulatory processes, and provide neuroprotective effects for child development (Balldin et al., 2018; Poslawsky et al., 2015; Provenzi et al., 2020; Tryphonopoulos and Letourneau, 2020). VF interventions include a diverse range of procedures and methodologies aimed at promoting positive parenting. They capitalize on allowing parents to observe themselves and their interactions with their infant “from the outside,” thereby facilitating mentalization and reflective functions (Leyton et al., 2019; Riva Crugnola et al., 2021). Although different theoretical and methodological VF approaches have been described (Provenzi et al., 2020), previous research highlighted benefits for child development and the quality of parent–child interaction in different clinical contexts and populations, including preterm infants and their caregivers (Barlow et al., 2016; Hoffenkamp et al., 2015). More recently, a clinical trial by Pisoni et al. (2021) highlighted long-term improvement in the developmental quotient scores of 24-month-old VPT infants of age following a VF intervention, adding to the evidence that remote video consultation may be an effective home care approach (Hägi-Pedersen et al., 2021; Suir et al., 2022).

Hyperscanning is a relatively recent approach to the study of brain-to-brain co-regulation in live interactive partners using different electrophysiological and neuroimaging techniques (Bi et al., 2023; Nguyen et al., 2020). By simultaneously recording multiple brains’ activity, hyperscanning allows the acquisition of neurophysiological measures of human dyadic or group-based neurophysiological coordination (Czeszumski et al., 2020). Among the different available techniques, EEG offers special advantages when the hyperscanning paradigm is applied to pediatric and even newborn populations. Wireless EEG devices are relatively non-invasive and allow for freedom of movement, making them ideal for studying mother–infant interactions in both laboratory and ecological settings during the first months of life. By using EEG-hyperscanning paradigms, Leong et al. (2017) highlighted how gaze direction during face-to-face interactions between adults and 8-month-old infants affects patterns of dyadic neural connectivity. Similarly, different patterns of theta power fluctuations were observed when 12-month-old infants play solo in the presence of the caregiver or when they actively interact together (Wass et al., 2018). More recently, the phase-locking values (PLVs) indicating the strength of brain-to-brain co-regulation in theta and alpha frequency bands were computed during 7-month-old infants’ interaction with the caregiver compared to an adult stranger (Endevelt-Shapira et al., 2021). The study reported greater inter-brain attunement when infants were interacting with the mother, despite the addition of maternal chemo-signals in the setting of infant–stranger interaction attenuated the significant difference.

As the field of parent–infant hyperscanning research is rapidly growing, the accumulating knowledge is contributing to a pivotal epistemic and theoretical shift in developmental neurosciences from a mono-personal account to a strongly relational perspective (Dumas, 2011). The application of hyperscanning paradigms to the study of parent–infant brain-to-brain co-regulation in at-risk and clinical pediatric populations holds promises to acquire innovative data on the mechanisms by which the early caregiving environment fosters and promotes child neurodevelopment (Provenzi et al., 2023). Moreover, as specific indices of brain-to-brain co-regulation become validated in typical development, they may also be embedded into a novel set of neurobehavioral markers to assess the benefit of early interventions. Such *translational hyperscanning* vision has been recently framed in the affective neuroscience literature (Provenzi et al., 2023), yet there is a lack of research on the brain-to-brain co-regulation of VPT infants and their parents and on the potentially beneficial neuroprotective effects of early parenting interventions in this population.

## Study AIMS

### General and specific aims

The *2-Brain Regulation to Achieve Improved Neuroprotection during Early Development* (2-BRAINED) research project is funded by the Italian Ministry of Health under the Ricerca Finalizzata 2021 program (research line: Giovani Ricercatori, project code: GR-2021-12375213). It is aimed to assess brain-to-brain co-regulation patterns in dyads of VPT infants and their caregivers and further explore how an early VF intervention may facilitate specific inter-brain regulatory indices.

The first specific aim (Aim 1) is to assess the presence of statistically significant differences in a set of brain-to-brain co-regulation indices—primarily, PLV measure—between dyads of caregivers and VPT compared to FT infants. Previous research gave evidence of specific markers of lower co-regulation in behavioral synchrony (Montirosso et al., 2010), physiological coupling (Feldman and Eidelman, 2007; Porges et al., 2019), and neuroendocrine attunement (Provenzi et al., 2019) in dyads of VPT infants during the first year of life. Consistently, we hypothesized that VPT infants and their caregivers would show less strong brain-to-brain co-regulation indices compared to dyads of FT counterparts at 9 months (corrected age for prematurity, CA).

The second specific aim (Aim 2) is to investigate the effect of an early VF intervention for parents of VPT infants on the selected indices of brain-to-brain co-regulation at 9 months CA. By comparing EEG-hyperscanning-derived indices of inter-brain coupling between dyads of VPT infants exposed to the VF intervention and dyads exposed to care as usual during the 3 months following NICU discharge, we hypothesize to describe greater brain-to-brain co-regulation in the former group.

### Additional exploratory aims

The longitudinal nature of this study and the possibility to collect a multi-layer set of data for what pertains to the neurobehavioral development of FT and VPT infants as well as the parenting environment during the first year of life allow us to set the stage for a number of exploratory analyses that will further guide future spin-off studies stemming from the 2-BRAINED research project. In this study, we highlight five main exploratory aims that appear relevant for future translational research in the field of affective neuroscience and developmental psychobiology.

First, the availability of behavioral and EEG physiological data from the 9-month interactive procedure (see below, *Study design and procedures*) will allow for the exploration of patterns of bio-neurophysiological coupling within dyads. Previous research produced limited evidence for the presence of correlations between specific interactive behaviors and EEG signaling during interactive tasks (Liu et al., 2018). Similarly, in VPT infants and their caregivers, the presence of a matched coupling or overlapping regulatory profiles between interactive behaviors and neuroendocrine or physiological oscillations is debated (Provenzi et al., 2019). This study will provide a suitable data setup to further explore the presence of significant coupling between interactive behaviors and neurophysiological brain activity in typically developing and at-risk pediatric populations.

Second, it will be possible to explore how brain-to-brain co-regulation in typically developing FT infants and their caregivers is affected by different dimensions that characterize infants’ development (e.g., sensory profile and temperament) and the caregiving environment (e.g., affective symptoms, parenting stress, and parent–infant bonding). It is well known that different behavioral indices of caregiver–infant co-regulation (e.g., matching, synchrony, and dyadic reparation (Provenzi et al., 2018)) are shaped by individual characteristics and contributions by parent and infant behavior. For instance, Fuertes et al. (2006) have suggested that infant temperament may play a critical role in the emergence of early attachment patterns and co-regulation of socio-emotional stress in full-term infants. Similarly, maternal affective symptoms may result in different dyadic organizations of critical behaviors signaling reciprocal attention and socio-emotional availability, such as gaze direction (Lotzin et al., 2015) and emotional cues (Bigelow et al., 2018).

Third, the role of sensory profile and environmental sensitivity to sensory inputs is recognized as an important contributor to child socio-emotional stress regulation (Greven et al., 2019; Lionetti et al., 2018). Previous research has highlighted how infants with diverse sensory profiles (e.g., sensation seekers or passive encoders) may exhibit differences in their resting state EEG activity (Pierce et al., 2021). Additionally, sensory reactivity in FT and VPT infants at 12 months has been found to be associated with later behavioral problems in toddlerhood (Maitre et al., 2020). The role of infants’ sensory profile in setting the stage for different gradients of brain-to-brain co-regulation is yet to be explored, and such investigation may shed light on genetic-informed individual differences in the early establishment of parent–infant relationship.

Fourth, consistent with the previous exploratory goal, it can be speculated that VPT infants—due to early adverse sensory stimulations during the NICU stay (Aita et al., 2013; Pineda et al., 2019)—may exhibit specifically altered profiles of sensory regulation compared to FT counterparts. Niutanen et al. (2020) recently conducted a review of the literature highlighting how VPT infants may exhibit abnormal regulation of sensory inputs with consequences for sensory-motor integration and stress regulation. As the sensory regulation profile of VPT infants may be partially learned from attempts to adapt to the NICU, the present study may also help identify how early alterations in the sensory environment influence the emergence of precocious forms of brain-to-brain co-regulation with the caregiver.

Finally, by collecting quantitative data on the parents’ experience of the NICU hospitalization—including both psychological stress and perceived support from the staff—it will be possible to estimate how caring for parents’ wellbeing during the NICU stay may promote later electrophysiological caregiver–infant attunement. Previous research has highlighted that mothers of VPT infants may exhibit lower sensitivity to their infants’ facial and bodily cues (Butti et al., 2018). However, their brain reactivity to emotional pictures of their own VPT infants appears heightened compared to that of FT infants’ mothers (Montirosso et al., 2017). These preliminary findings suggest that the brains of VPT infants’ caregivers may process interactive-salient stimuli differently, potentially influenced by the stressful experience of NICU hospitalization and early parent–infant separation. The present study will allow us to study how the brain activity of VPT infants’ mothers processes relevant social cues during real-life face-to-face interactions, further contributing to understanding how caregivers’ brain adapts to preterm birth and hospitalization. In this context, NICU-related stress and perceived support from staff could be considered potential moderators of the caregivers’ EEG activity when interacting with their VPT infant—with relevant consequences for the observed brain-to-brain co-regulation.

## Methods and procedures

### Study design and procedures

The 2-BRAINED project is a randomized-controlled trial (RCT) with three arms. The first arm includes VPT infants and their caregivers randomly allocated to the intervention arm (VPT-VF). The second arm includes VPT infants and their caregivers randomly allocated to the care as usual arm (VPT-CU). This arm will act as a control group matched to preterm conditions of VPT-VF. The third arm includes FT infants and their caregivers and will act as an additional control group unmatched by preterm conditions. Both VPT-CU and FT arms will receive no VF intervention.

#### Population, enrollment, and arm allocation

VPT and FT infants will be enrolled at birth by contacting their caregivers within the first 48 h after delivery. Informed consent will be obtained. VPT infants will be considered eligible in the presence of the following conditions, as reported in medical charts: gestational age below 35 weeks, absence of major brain lesions as documented by cerebral ultra-sound, no neuro-sensory deficits including retinopathy of prematurity (ROP) equal or above stage 2, absence of genetic syndrome, or malformations involving the central nervous system. FT infants will be considered eligible if they meet the following conditions: a gestational age of 37 weeks or more, are healthy, and show no evident signs of neurodevelopmental risk or morbidities. For both groups, exclusion criteria will include single-parent families, parental age under 18 years, lack of Italian language mastery, and the presence of documented psychiatric disorders.

#### Study timeline

**Figure 1:**
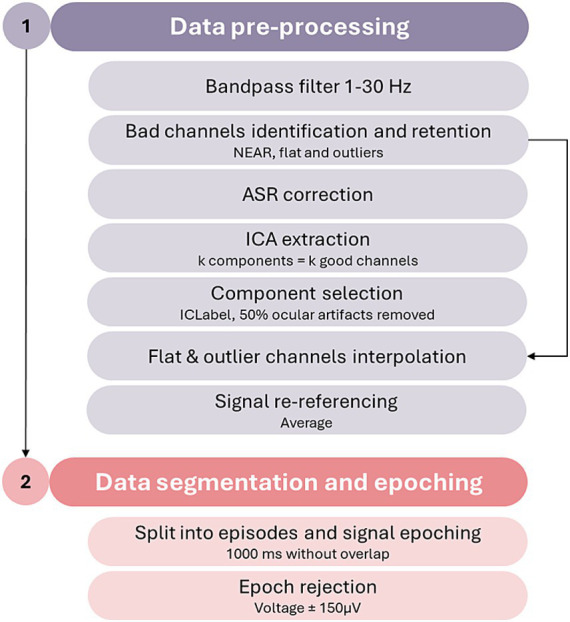
EEG data pre-processing pipeline. NEAR, Neonatal EEG Artifact Removal; ASR, artifact subspace reconstruction; ICA, independent component analysis.

The 2-BRAINED study features five data collection waves (see Figure 1). The VF intervention is delivered after wave T1 (NICU discharge) and before wave T2 (3 months CA) to subjects allocated to the VPT-VF arm. The EEG-hyperscanning task will occur for all subjects at wave T4 (9 months CA) and will feature the videotaping of mother–infant interaction according to a modified Face-to-Face Still-Face (FFSF) procedure (Tronick et al., 1978) and the simultaneous EEG data collection from both the interactive partner. At each wave, parents will receive questionnaires by email using REDCap.^1^

#### VF intervention

##### Remote videotaping

Before (T1) and after (T2) the VF intervention sessions for participants allocated to VPT-VF—and at the same timepoints for participants allocated to VPT-CU—a 15-min mother–infant interaction will be videotaped remotely. Before videotaping, mothers will be asked to position the webcam or smartphone to have the widest possible view of the play area and see the entire body of both the mother and the infant. The interaction paradigm includes 10-min unrestrained face-to-face play followed by a 6-min FFSF procedure (Tronick et al., 1978) as described here: During the 2-min Play episode, mothers will be asked to play with the infant as they usually do (e.g., the infant can stay in an infant seat or on a carpet); during the 2-min Still-Face episode, mothers will be asked to interrupt any communication and maintain a still, poker face while keeping eye-contact with their infant; unconstrained interaction will be resumed during the 2-min Reunion episode.

##### Intervention details

The remote VF intervention has been adapted according to previous research from our group (Grumi et al., 2021). It comprises six weekly 1-h sessions organized in two subsequent phases: four *sharing the focus* sessions and two *integration* sessions. *Sharing the focus* sessions are dedicated to the discussion between the psychologist and the mother of specific themes related to parenting and parent–infant interaction: physical stimulation, responsiveness, teaching, and parenting experience (see Table 1). During these sessions, a purposively trained psychologist invites the mother to jointly review and discuss brief clips obtained from the pre-intervention videotaped interaction, usually starting from potential curiosity, comments, or requests from the mother herself. The goal of the *sharing of the focus* sessions is to develop insights about the infants’ behavioral signals, how to respond contingently and appropriately, how to promote emotion regulation, and how to sustain cognitive and behavioral achievements. In the subsequent two *integration* sessions, the mother plays with the infant while the psychologist provides guidance based on insights co-developed during the previous four sessions. The goal is to promote a pragmatic translation of the insights into interactive skills.

**Table 1:** Description of the thematic focus of the four *sharing the focus* sessions of the video-feedback (VF) intervention.

Thematic cluster	Key topic	Goal
Sensory stimulation and regulation	Sensory channels	Highlighting infant preference or avoidance of specific sensory channels and stimuli.
	Intensity of stimulation	Regulating intensity of physical stimulation and understanding infant sensory thresholds.
	Affective social touch	Facilitating parental affective touch to promote infant state regulation, postural stability, and attention orientation.
Nurturing and sensitive caregiving	Parental sensitivity	Promoting parental perception, interpretation, and appropriate responsiveness to infant communicative signals.
	Sense of agency	Detecting and supporting the infant’s interactive initiatives (e.g., vocalizations and attention orienting).
	Exploration and safety	Supporting the infant exploration of the environment and building safety and trust in parental secure base.
	Rhythm and reparation	Facilitating the emergence of a proto-conversational rhythm in the dyad and supporting reparative actions of interactive perturbations.
Cognitive sensitivity and scaffolding	Attention skills	Supporting and scaffolding infant sustained and focused attention to the physical and social environment.
	Modeling and guidance	Providing a model to foster observational learning and the functional use of tools and toys.
	Proximal development zone	Improving caregiver awareness of the cognitive abilities of the infant to make appropriate play proposals and support infant emerging abilities.
Parenting experience and mental state	Mind-mindedness	Improving awareness about parental representations of the infant mind and keeping high levels of curiosity about infant behaviors.
	Self-care and self-regulation	Highlighting the importance of parental psychological wellbeing and reflective functions; promoting parental psychological self-care and compassion.
	Self-efficacy	Strengthening the caregiver’s sense of efficacy as a parent and nourishing trust in the parent’s own experience and mental representations of the infant.

#### FFSF procedure

At 9 months (CA for VPT participants), mothers and infants will take part in a FFSF procedure in the laboratory. The FFSF will include three episodes: During the Play episode (2 min), mothers and infants will interact face-to-face avoiding the use of toys and pacifier; during the Still-Face episode (1 min), mothers will be asked to interrupt any communication toward the infant, to maintain a still, poker face, while maintaining eye-contact; and during the Reunion episode (2 min), unconstrained interaction will be resumed. The procedure has been previously adopted to assess biobehavioral dimensions (Provenzi et al., 2019; Provenzi et al., 2017) and physiological underpinnings (Montirosso et al., 2010; Mantis et al., 2014) of socio-emotional stress regulation in VPT infants. The entire procedure will be videotaped for the offline coding of specific maternal and infant interactive behaviors (see *Measures* for details).

#### Neurophysiological procedures

EEG data acquisition will occur at 500 Hz sampling frequency during the 9-month FFSF procedure employing the Smarting Pro (mBrainTrain, Belgrade, Serbia) system equipped with two 32-channel EEG caps featuring wireless Bluetooth connection between the amplifiers and the mBrainTrain Streamer software installed on two separate laptops. The laptops receiving data will be linked to each other via a network cable to ensure synced data collection. The use of wireless EEG caps will allow greater flexibility and comfort for participating dyads.

Upon arrival, the infant will be familiarized with the setting: A play mat and toys will be available to aid in acclimatization to the environment. The researchers will debrief parents with a comprehensive explanation of the study’s aims and procedures. Cap sizes will be selected to fit participants’ head circumference. The caps fitting process will commence with the caregiver to ensure greater infant comfort and familiarity with the equipment. The conductive gel will be applied to optimize signal conductivity and minimize artifacts.

### Measures

Demographic (e.g., parental age, parental job, and parental educational level), neonatal (e.g., gestational age, birth weight, and Apgar score), and clinical variables (e.g., NICU length of stay and minor morbidities) will be obtained from medical charts. Parent-report questionnaires are summarized and described in Table 2. As for behavioral coding purposes, the videotapes obtained from two cameras during the lab FFSF procedure will be edited offline using Movavi Video Suite 2020 software and a single synced video showing both frontal views of the caregiver and the infant’s face, hands, and torso will be produced. Videos will be micro-analytically coded for infants’ and caregivers’ target interactive behaviors according to an adaptation of the Parent–Infant Coding Scheme (PICS, Version 4.0; Brambilla et al., 2023) as reported in Table 2. PICS codes will be computed as a percentage of time for each FFSF episode (Table 3).

**Table 2:** Details of questionnaires included in the study.

Construct	Questionnaire	Reference	Item N	Likert scale	Description	Study wave(s)
Parental NICU-related stress	Parental Stressor Scale—NICU (PSS-NICU)	Miles et al. (1993)	46	5-point	Three main factor scores representing stress related to infants’ appearance, environmental sights and sounds, and parental role alteration	T1 (only VPT)
Sensory profile	Sensory Profile-2 (SP-2)	Dunn (2014)	54	5-point	The infant version (0–6 months) identifies 5 sensory patterns. The toddler version (7–35 months) identifies four sensory patterns.	T2, T3, T4
Anxiety symptoms	State–Trait Anxiety Inventory (STAI-Y)	Spielberger et al. (1983)	40	4-point	One trait score representing a tendency to feel anxiety and one state score representing the present levels of anxiety	T2, T4
Depression symptoms	Beck Depression Inventory (BDI-II)	Beck et al. (1996)	21	4-point	Global score representing a quantitative appreciation of the severity of symptoms of depression	T2, T4
Parenting stress	Parenting Stress Index—Short Form (PSI-SF)	Abidin et al. (2006)	36	5-point	Three subscale scores addressing parental distress, parent–child dysfunctional interaction, and stress related to difficult child behavior. A global score is also obtained.	T2, T3, T4
Temperament	Infant Behavior Questionnaire-Revised (IBQ-R) very short form	Gartstein and Rothbart (2003)	37	7-point	Three subscale factor scores addressing negative affectivity, surgency, and regulatory capacity.	T2, T3, T4

VPT, very preterm; NICU, neonatal intensive care unit.

**Table 3:** Selection of codes from the Parent-Infant Coding Scheme (PICS, Version 4.0).

Variable	Levels	Description
A. Both interactive partners		
Emotional state	Negative	Clear display of negative emotionality (e.g., eyes, mouth, general movements of the face or the body, and other vocal or non-vocal signals) including fussing and crying.
	Neutral	No clear display of negative or positive emotionality.
	Positive	Clear display of positive emotionality (e.g., eyes, mouth, general movements of the face or the body, and other vocal or non-vocal signals) including smiles and laughs.
Gaze direction	Face-directed	Attention focus is on the interactive partner’s face
	Object-directed	Attention focus is on the interactive partner body (e.g., hands and torso) or other objects.
	Avoiding	The subject is actively avoiding eye contact as displayed by head and body movements/posture.
Approach/withdrawal	Withdrawal	Evident leaning backward and/or turning the head away to avoid interaction
	Neutral	No evident backward or forward movements.
	Approach	Evident leaning forward and/or reaching forward to engage in interactive behaviors.
B. Parental-specific codes		
Vocal inputs	No voice	No vocal productions.
	Negative	Vocal comments that convey explicit critique or rejection of infants’ behaviors or state.
	Pragmatic	Vocal comments that are finalized to modify or instruct the interactive partner’s cognitive state, such as requests, attention-getting, and explanations.
	Social	Vocal comments that convey playful and social engagement such as singing, laughing, and playing nursery rhymes.
	Nurturing	Vocal comments that express appreciation or acceptance of infants’ behaviors or state or are finalized to soothe infants’ stress. These also include mind-related comments (e.g., “you think” and “you want”) and mirroring of infants’ communicative bids.
Tactile inputs	No touch	No tactile stimulations.
	Negative	Tactile stimulations that clearly appear intrusive and/or provoke or increase a negative emotionality state in the interactive partner.
	Pragmatic	Tactile stimulations that are finalized to modify or instruct the interactive partner postures or movements in the environment, such as holding, shadowing, and attention-getting.
	Social	Tactile stimulations that convey playful and social engagement such as tickling, squeezing, and any other appropriate entertaining tactile stimulations that are fast-paced, dynamic, repetitive, and/or characterized by quick cinematic features.
	Nurturing	Tactile stimulations that are finalized to soothe or regulate the behavioral state of the interactive partners. These include stroking, kissing, massaging, and any other appropriate tactile stimulations with clear regulatory functions and conveying a sense of affective closeness.

The complete coding manual is available upon request to the corresponding author.

### Plan of EEG data elaboration

#### Pre-processing pipeline

**Figure 2:**
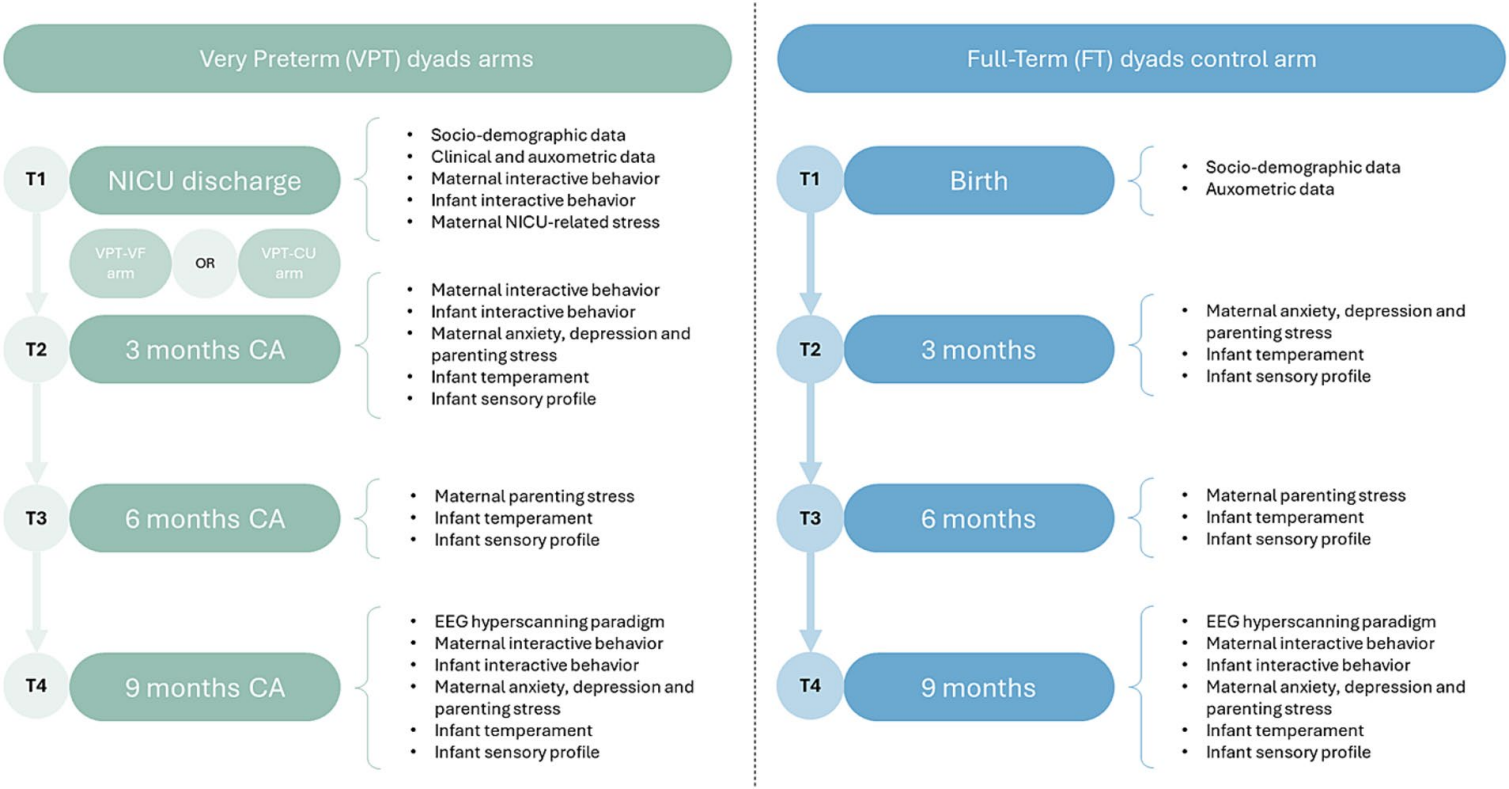
Study design. NICU, neonatal intensive care unit; VF, video-feedback intervention; CU, care as usual; CA, corrected age.

Dyadic EEG data will be pre-processed with a fully automated pipeline built using the MATLAB-based (The MathWorks Inc., 2024) interacting toolbox EEGLAB (Delorme and Makeig, 2004). A brief description of the main pre-processing steps is available in Figure 2.

The parent and infant signals will be pre-processed separately with the same steps and parameters for both. First, data filtering will be performed with the application of a bandpass 1–30 Hz filter as the planned analyses (see below) will be conducted on the lower (theta and alpha) frequency bands. Subsequently, flat and outlier channels will be detected using the Neonatal EEG Artifact Removal (NEAR) plugin (Kumaravel et al., 2022) and retained (i.e., put in a separate temporary matrix) for later interpolation. Dyads in which at least one of the members displays a signal with more than 15% (*N* > 5) of flagged channels will be excluded from further analyses. The EEG signal from all non-flagged channels will undergo noise correction through the artifact subspace reconstruction (ASR; Chang et al., 2020), with burst criterion (k) set at 10; subsequently, analysis of the components of the signal will be performed through the independent component analysis (ICA; runica function with default settings), producing as many components as the number of good channels. The resulting components will be then classified through the ICLabel (Pion-Tonachini et al., 2019) plugin. Every component flagged as having a 50% or more probability of being an ocular artifact will be rejected. At this stage, the matrix containing the flat and outlier channels will be re-merged with the EEG matrix, and the bad channels will be interpolated through spherical interpolation using the pre-processed signal. The signal will be then re-referenced to the average signal of the channels and split into three different sets containing each phase of the experimental procedure (Play, Still-Face, and Reunion): Each of these sets will be subsequently segmented into 1,000 ms epochs avoiding overlaps. Bad data segments containing residual artifacts in each of the three phases will be identified. All segments in which at least one of the target channels used for estimates of dyadic co-regulation (see below) displays a voltage exceeding ±150 μV (Debnath et al., 2020) will be marked as rejected. The rejected epochs in the infant’s and parent’s signals will be merged to obtain the final pool of rejected epochs for the dyad. This ensures that all the rejected epochs for one interactive member of the dyad will be similarly mirrored for the other partner. Dyadic data will undergo further analyses if their signal contains at least 30 good epochs in both the Play and Reunion phases; if this criterion is not met, their signal will undergo manual epoch rejection performed by an expert EEG coder. In case after the manual epoch rejection, the dyad will result having less than 30 good merged epochs in at least one of the FFSF episodes, the signal will be excluded from further analyses.

#### Estimating indices of inter-brain co-regulation

Several inter-brain synchronization indices have been proposed so far to estimate the coupling between two brains (Czeszumski et al., 2020). Since there is still debate on the appropriateness of each inter-brain synchronization measure, we plan to compute and compare several indices (see Table 4). To further check for spurious findings and gather stronger evidence that the obtained co-regulation estimations are not artifact production, we will compare the synchronization indices obtained from the real dyads to surrogate data generated by randomly pairing mothers and infants from different dyads.

**Table 4:** Indices of inter-brain co-regulation adopted in the 2-BRAINED study.

Index	Description	Notes	Reference
Phase-Locking Value (PLV)	Frequency-specific transients of phase locking independent of amplitude. The value ranges from 0 to 1: values closer to 0 indicate random signals with unsynchronized phases; values closer to 1 indicate stronger coupling between the two signals.	While previous studies focused mainly on infant frequency bands, cross-frequency PLV indices will be obtained for the purposes of the 2-BRAINED study.	Lachaux et al. (1999); Canolty and Knight (2010)
Imaginary Coherence (ICoh)	Computed through spectral density (power) of each participant and cross-spectral density between them to estimate the average phase difference and consistency of phase difference synchronization.	ICoh is expressed as a complex number: the real part represents how much the coherence is driven by instantaneous interactions; the imaginary part shows how much the coherence is based on lagged interactions.	Dikker et al. (2021); Turk et al. (2022)
Amplitude-Amplitude Coupling (AAC)	Expressed as Pearson’s correlation coefficient between normalized power time courses of the two signals.	Amplitude coupling was suggested as a valid alternative to phase coupling for three main reasons: amplitude changes are more easily estimated; amplitude modulations are more extensively characterized across EEG studies; amplitude modulations are more sensitive to neural coupling phenomena non-detectable with other phase-related measures.	Haresign et al. (2022); Koul et al. (2023)

The computation of synchronization measures will preferentially occur considering homologous channels for the sake of interpretability and computational costs. However, as an exploratory analysis, we will also compute synchronization between non-homologous channels because we can hypothesize that synchronization tasks between mother and infant can involve different brain areas in the two actors (Endevelt-Shapira et al., 2021).

Regarding the frequency bands on which the synchronization measures will be computed, we will mainly consider alpha and theta. Indeed, these frequencies have been found to be involved in parent–infant social tasks, with theta fluctuations linked to changes in shared attention during joint play of parent and infant (Wass et al., 2018), enhancement of alpha and theta power linked to changes in directed gaze (Leong et al., 2017), and fluctuations in alpha band linked to changes in emotional states of mother and child (Santamaria et al., 2020).

Moreover, since we are interested in the dynamic evolution of brain-to-brain synchronization, we plan to evaluate the trend of each synchronization index over time (i.e., over the epochs). In particular, we are interested in the change between an asynchronous to a synchronous state, which is defined as reparation. Reparation is a dyadic process in which unmatched dyadic states are transformed into matched dyadic states. We will compute the rate and the latency of reparation considering the EEG synchronization indices as previously performed in synchronized behaviors assessment (Provenzi et al., 2015).

### Statistical power and sample size estimates

The sample size has been estimated according to over-arching Aim 2, setting parameters as follows: medium effect size, *f* = 0.25, *alpha* = 0.05, *beta* = 0.20, number of groups = 3 (VPT-VF, VPT-CU, and FT). The procedure yielded a total sample size of 159 subjects (53 subjects per RCT arm). Nonetheless, considering the longitudinal nature of the study and the attrition rate related to EEG tasks with infants, an oversampling of *n* = 80 (~ +50%) subjects per RCT arm was planned to secure the minimum sample size for appropriately powered statistical analyses.

### Plan of statistical analyses

#### Preliminary analyses

##### Specific aim 1

General linear models (GLMs) will be carried out to compare VPT and FT dyadic brain-to-brain co-regulation indices during the experimental procedure phases. Theoretically relevant (e.g., gestational age) and statistically identified (e.g., any variable significantly linked with the outcome variables) confounding variables will be controlled for in the analytical model.

##### Specific aim 2

Separate analyses of variance (ANOVA) will be used with dyadic brain-to-brain co-regulation indices as the dependent variable and groups (FT, VPT-VF, and VPT-CU) as the independent variable. Theoretically relevant (e.g., gestational age) and statistically identified (e.g., any variable significantly linked with the outcome variables) confounding variables will be controlled for in the analytical model.

##### Additional aims

Models to track early developmental trajectories will be estimated in Mplus by latent class growth analysis with inter-individual variations in time of assessment and mixed-effect linear models with repeated measures to assess group differences in rates of temperament, emotional, and sensory profiles.

## Discussion

The present protocol describes an RCT study that aims to assess the benefits of an early post-discharge video-feedback intervention to enhance and promote both parental and VPT infants’ outcomes. By collecting dual-source EEG data in a hyper-scanning paradigm and from face-to-face real-time interactions between parents and infants, the study also aims at providing estimations of the effects of such intervention not only for the individual adjustment of caregivers and infants but also for the emergence of dyadic co-regulatory biobehavioral processes. Such co-regulation profiles are meant to be critical indicators of a nurturing caregiving environment during the first months of life fostering affective wellbeing and stress resilience (Feldman, 2020; Levy and Feldman, 2019).

### Sources of bias and mitigation strategies

The heterogeneity of VPT infants’ conditions should not be underestimated. Even in the absence of severe comorbidities and brain injuries, the experience of NICU hospitalization might be very different for each infant and their parents. To avoid extreme variations, the gestational age range will be constrained between 28 and 35 weeks. Moreover, stress related to the NICU environment will be evaluated and quantified with a well-validated questionnaire (Miles et al., 1993). Selection issues might affect random allocation plans in RCT arms. The allocation to VPT-VF and VPT-CU arms will occur by using an automatically generated list of binary codes that will be consecutively matched with the enrolled families across consecutive sampling. This will reduce the risk of self-selection. To further avoid confounding by infant sex and assure sex distribution balancing, the random allocation will be stratified by infant sex by *post-hoc* controls every 20 enrollments. Parental gender will also be unconstrained, inviting the primary caregiver—and not explicitly the mother—to participate in the study, VF intervention, and observational procedures. EEG procedural steps and artifacts might easily result in the loss of subjects in a longitudinal study; a 50% oversampling was planned to achieve the minimum sample size for adequately powered statistical analyses.

### Expected results and impact

The project represents a translational application of the emerging field of hyper-scanning in developmental neuroscience (Provenzi et al., 2023). As neuroscience is moving toward a radical shift in considering interpersonal exchanges as the primary unit of analysis and observation (Leong et al., 2019; Hoehl and Markova, 2018), clinical applications are meant to be implemented to innovate healthcare. Previous proposals have been advanced to apply such a bi-personal neuroscientific approach to the field of adult psychiatry (Saul et al., 2022) and child development (Nguyen et al., 2020).

In this study, we aim to innovate the field of family-centered care in pediatric settings by embedding a cutting-edge approach to the study of parent–infant interaction and co-regulation processes into well-validated approaches to parental support and child development promotion. As the video-feedback intervention is well-acknowledged for its beneficial implications for parental wellbeing, child development, and quality of the early parent–child relationship (Fukkink et al., 2011; Montirosso et al., 2020), it represents an élite clinical setting to test the advantages of new neuroscientific-inspired metrics that specifically focus on the assessment of brain-to-brain co-regulatory processes. In this context, the present study has multifaceted implications.

From a scientific perspective, this study will provide first-of-a-kind quantitative estimations of inter-brain coupling and co-regulation in a sample of VPT infants and their caregivers. While previous research has highlighted functional and structural alterations in VPT infants’ brains (Dereymaeker et al., 2017; Hüppi et al., 1998; Pittet et al., 2019), very little is known about VPT neurophysiological functioning in real-life settings. Moreover, the study will provide insights into “how much” inter-brain synchrony should be expected in typical and atypical developmental trajectories. As medium levels of attunement and matching have been suggested to be optimal in terms of behavioral co-regulation during the first months (Provenzi et al., 2016), similar expectations appear to be plausible for what pertains to inter-brain coupling.

From a clinical point of view, the 2-BRAINED project is expected to produce evidence of the efficacy of an early intervention for VPT infants and their parents that is delivered after NICU discharge. This is a critical window for continuity of care as parents transition from potentially high-quality family-centered care during NICU stay to a lack of appropriate and tailored support at home (Lundqvist et al., 2019; Provenzi et al., 2016). From this perspective, this study will explore the efficacy of an intervention aimed at granting continuity of care from the hospital to the house, extending and empowering family-centered care for VPT infants’ parents.

From a translational neuroscience perspective, the study will also offer an unprecedented opportunity to obtain first-hand dyadic neurophysiological target outcomes of well-validated early family-centered VF intervention. While the road to developing qualitative and quantitative measures of the effectiveness and efficacy of such family-centered interventions is yet to be fully implemented, the integration of behavioral, self-report, and neurobiological markers is promising for future advances.

### Patient and public involvement

Active engagement of families will be pursued through public-dedicated web/social communications, digital content, and newsletters describing the achieved goals and implications of the study. Parental associations and professional orders will be engaged through online webinars to capitalize on the data obtained from the present study to further fuel a culture of family-centered care for preterm infants and their parents.

## Ethics and dissemination

### Ethics, privacy, and data management

The Ethics Committee Pavia and collaborating partners granted approval for the study on 16 February 2023 (protocol number: 0008588/23) and officially launched on 24 April 2023 (GR-2021-12375213). All procedures align with the ethical principles outlined in the Declaration of Helsinki for research involving human subjects, ensuring no harm to participants. The study intervention offers additional opportunities for families without altering standard mother–infant care programs. Infants will undergo planned diagnostic and therapeutic interventions at the child neurology and psychiatric unit IRCCS Mondino Foundation, Pavia, Italy, and the Scientific Institute IRCCS *E. Medea*, Bosisio Parini, Italy.

Data management will occur in accordance with the General Data Protection Regulation (Regulation 2016/279, commonly known as GDPR), to guarantee the privacy and the security of the gathered data. In this sense, all the infants’ parents will sign an informed consent module after that the study’s aims and modalities will be clearly explained and eventual doubts will be solved. Each subject will be assigned a code, and data will be stored in a pseudonymized form. After the period of conservation (25 years), data will be made completely anonymous. In line with the open science principle, the anonymized data collected for the study will be published on a publicly accessible “repository” (i.e., Zenodo) to promote the dissemination of research results with a view to furthering the research itself and the scientific community.

### Dissemination

The dissemination strategy involves presenting findings at national and international scientific meetings, publishing in developmental psychology journals, and engaging in outreach activities with families and healthcare specialists. This aims to promote early family-centered intervention and share insights with the wider public.

## Footnotes

^1^ https://www.project-redcap.org/

## References

[ref90012] Abidin R. Flens J. R. Austin W. G. (2006). The parenting stress index. Lawrence Erlbaum Associates Publishers.

[ref1] Aita M. Johnston C. Goulet C. Oberlander T. F. Snider L. (2013). Intervention minimizing preterm infants’ exposure to NICU light and noise. Clin. Nurs. Res. 22, 337–358. doi: 10.1177/1054773812469223, PMID: 23275433

[ref2] Balldin S. Fisher P. A. Wirtberg I. (2018). Video feedback intervention with children: a systematic review. Res. Soc. Work. Pract. 28, 682–695. doi: 10.1177/1049731516671809

[ref3] Barlow J. Sembi S. Underdown A. (2016). Pilot RCT of the use of video interactive guidance with preterm babies. J. Reprod. Infant Psychol. 34, 511–524. doi: 10.1080/02646838.2016.1217404

[ref4] Beam A. L. Fried I. Palmer N. Agniel D. Brat G. Fox K. . (2020). Estimates of healthcare spending for preterm and low-birthweight infants in a commercially insured population: 2008–2016. J. Perinatol. 40, 1091–1099. doi: 10.1038/s41372-020-0635-z, PMID: 32103158 PMC7314662

[ref9001] Beck A. T. Steer R. A. Brown G. K. (1996). “Beck depression inventory–II.” Psychological assessment. Unpublished manual.

[ref5] Bi X. Cui H. Ma Y. (2023). Hyperscanning studies on interbrain synchrony and child development: a narrative review. Neuroscience 530, 38–45. doi: 10.1016/j.neuroscience.2023.08.035, PMID: 37657749

[ref6] Bigelow A. E. Beebe B. Power M. Stafford A.-L. Ewing J. Egleson A. . (2018). Longitudinal relations among maternal depressive symptoms, maternal mind-mindedness, and infant attachment behavior. Infant Behav. Dev. 51, 33–44. doi: 10.1016/j.infbeh.2018.02.006, PMID: 29567547

[ref9002] Brambilla M. Grumi S. Manfredini V. Pettenati G. Provenzi L. (2023). Parent-Infant Coding System, PICS. Version 4.0, Unpublished manual.

[ref7] Burke S. (2018). Systematic review of developmental care interventions in the neonatal intensive care unit since 2006. J. Child Health Care 22, 269–286. doi: 10.1177/1367493517753085, PMID: 29328777

[ref8] Butti N. Montirosso R. Borgatti R. Urgesi C. (2018). Maternal sensitivity is associated with configural processing of infant’s cues in preterm and full-term mothers. Early Hum. Dev. 125, 35–45. doi: 10.1016/j.earlhumdev.2018.08.018, PMID: 30199717

[ref9003] Canolty R. T. Knight R. T. (2010). The functional role of cross-frequency coupling. Trends Cogn Sci. 14, 506–515. 20932795 10.1016/j.tics.2010.09.001 PMC3359652

[ref9] Caporali C. Pisoni C. Gasparini L. Ballante E. Zecca M. Orcesi S. . (2020). A global perspective on parental stress in the neonatal intensive care unit: a meta-analytic study. J. Perinatol. 40, 1739–1752. doi: 10.1038/s41372-020-00798-6, PMID: 32901116

[ref10] Chang C.-Y. Hsu S.-H. Pion-Tonachini L. Jung T.-P. (2020). Evaluation of artifact subspace reconstruction for automatic artifact components removal in Multi-Channel EEG recordings. IEEE Trans. Biomed. Eng. 67, 1114–1121. doi: 10.1109/TBME.2019.2930186, PMID: 31329105

[ref11] Cong X. Wu J. Vittner D. Xu W. Hussain N. Galvin S. . (2017). The impact of cumulative pain/stress on neurobehavioral development of preterm infants in the NICU. Early Hum. Dev. 108, 9–16. doi: 10.1016/j.earlhumdev.2017.03.003, PMID: 28343092 PMC5444300

[ref12] Czeszumski A. Eustergerling S. Lang A. Menrath D. Gerstenberger M. Schuberth S. . (2020). Hyperscanning: a valid method to study neural inter-brain underpinnings of social interaction. Front. Hum. Neurosci. 14:39. doi: 10.3389/fnhum.2020.00039, PMID: 32180710 PMC7059252

[ref13] Debnath R. Buzzell G. A. Morales S. Bowers M. E. Leach S. C. Fox N. A. (2020). The Maryland analysis of developmental EEG (MADE) pipeline. Psychophysiology 57:e13580. doi: 10.1111/psyp.13580, PMID: 32293719 PMC12758016

[ref14] Delorme A. Makeig S. (2004). EEGLAB: an open source toolbox for analysis of single-trial EEG dynamics including independent component analysis. J. Neurosci. Methods 134, 9–21. doi: 10.1016/j.jneumeth.2003.10.009, PMID: 15102499

[ref15] Dereymaeker A. Pillay K. Vervisch J. De Vos M. Van Huffel S. Jansen K. . (2017). Review of sleep-EEG in preterm and term neonates. Early Hum. Dev. 113, 87–103. doi: 10.1016/j.earlhumdev.2017.07.003, PMID: 28711233 PMC6342258

[ref9004] Dikker S. Michalareas G. Oostrik M. Serafimaki A. Kahraman H. M. Struiksma M. E. . (2021). Crowdsourcing neuroscience: inter-brain coupling during face-to-face interactions outside the laboratory. NeuroImage, 227:117436. 33039619 10.1016/j.neuroimage.2020.117436

[ref16] Dumas G. (2011). Towards a two-body neuroscience. Commun. Integ. Biol. 4, 349–352. doi: 10.4161/cib.4.3.15110, PMID: 21980578 PMC3187906

[ref9005] Dunn W. (2014). Sensory Profile 2 manual. San Antonio, TX: Pearson.

[ref17] Endevelt-Shapira Y. Djalovski A. Dumas G. Feldman R. (2021). Maternal chemosignals enhance infant-adult brain-to-brain synchrony. Sci. Adv. 7:eabg6867. doi: 10.1126/sciadv.abg6867, PMID: 34890230 PMC8664266

[ref18] Feldman R. (2006). From biological rhythms to social rhythms: physiological precursors of mother-infant synchrony. Dev. Psychol. 42, 175–188. doi: 10.1037/0012-1649.42.1.175, PMID: 16420127

[ref19] Feldman R. (2020). What is resilience: an affiliative neuroscience approach. World Psychiatry 19, 132–150. doi: 10.1002/wps.20729, PMID: 32394561 PMC7215067

[ref20] Feldman R. Eidelman A. I. (2007). Maternal postpartum behavior and the emergence of infant–mother and infant–father synchrony in preterm and full-term infants: the role of neonatal vagal tone. Dev. Psychobiol. 49, 290–302. doi: 10.1002/dev.20220, PMID: 17380505

[ref21] Fuertes M. Santos P. L. D. Beeghly M. Tronick E. (2006). More than maternal sensitivity shapes attachment: infant coping and temperament. Ann. N. Y. Acad. Sci. 1094, 292–296. doi: 10.1196/annals.1376.037, PMID: 17347364

[ref22] Fukkink R. G. Trienekens N. Kramer L. J. C. (2011). Video feedback in education and training: putting learning in the picture. Educ. Psychol. Rev. 23, 45–63. doi: 10.1007/s10648-010-9144-5

[ref9006] Gartstein M. A. Rothbart M. K. (2003). Studying infant temperament via the revised infant behavior questionnaire. Infant Behav. Dev. 26, 64–86.

[ref23] Greven C. U. Lionetti F. Booth C. Aron E. N. Fox E. Schendan H. E. . (2019). Sensory processing sensitivity in the context of environmental sensitivity: a critical review and development of research agenda. Neurosci. Biobehav. Rev. 98, 287–305. doi: 10.1016/j.neubiorev.2019.01.009, PMID: 30639671

[ref24] Grumi S. Borgatti R. Provenzi L. (2021). Supporting parenting at home-empowering rehabilitation through engagement (SPHERE): study protocol for a randomised control trial. BMJ Open 11:e051817. doi: 10.1136/bmjopen-2021-051817, PMID: 34907057 PMC8672000

[ref25] Hägi-Pedersen M.-B. Kronborg H. Norlyk A. (2021). Knowledge of mothers and fathers’ experiences of the early in-home care of premature infants supported by video consultations with a neonatal nurse. BMC Nurs. 20:54. doi: 10.1186/s12912-021-00572-9, PMID: 33827561 PMC8028708

[ref9007] Haresign I. M. Phillips E. A. M. Whitehorn M. Goupil L. Noreika V. Leong V. . (2022). Measuring the temporal dynamics of inter-personal neural entrainment in continuous child-adult EEG hyperscanning data. Dev. Cogn. Neurosci. 54:101093. 35248820 10.1016/j.dcn.2022.101093 PMC8899232

[ref26] He F. B. Axelin A. Ahlqvist-Björkroth S. Raiskila S. Löyttyniemi E. Lehtonen L. (2021). Effectiveness of the close collaboration with parents intervention on parent-infant closeness in NICU. BMC Pediatr. 21:28. doi: 10.1186/s12887-020-02474-2, PMID: 33430816 PMC7798198

[ref27] Hoehl S. Markova G. (2018). Moving developmental social neuroscience toward a second-person approach. PLoS Biol. 16:e3000055. doi: 10.1371/journal.pbio.3000055, PMID: 30543620 PMC6292561

[ref28] Hoffenkamp H. N. Tooten A. Hall R. A. S. Braeken J. Eliëns M. P. J. Vingerhoets A. J. J. M. . (2015). Effectiveness of hospital-based video interaction guidance on parental interactive behavior, bonding, and stress after preterm birth: a randomized controlled trial. J. Consult. Clin. Psychol. 83, 416–429. doi: 10.1037/a0038401, PMID: 25486375

[ref29] Hüppi P. S. Maier S. E. Peled S. Zientara G. P. Barnes P. D. Jolesz F. A. . (1998). Microstructural development of human newborn cerebral white matter assessed in vivo by diffusion tensor magnetic resonance imaging. Pediatr. Res. 44, 584–590. doi: 10.1203/00006450-199810000-00019, PMID: 9773850

[ref30] Ionio C. Ciuffo G. Landoni M. (2021). Parent-infant skin-to-skin contact and stress regulation: a systematic review of the literature. Int. J. Environ. Res. Public Health 18:4695. doi: 10.3390/ijerph18094695, PMID: 33924970 PMC8124223

[ref31] Jean A. D. L. Stack D. M. (2012). Full-term and very-low-birth-weight preterm infants’ self-regulating behaviors during a still-face interaction: influences of maternal touch. Infant Behav. Dev. 35, 779–791. doi: 10.1016/j.infbeh.2012.07.023, PMID: 22982279

[ref9008] Koul A. Ahmar D. Iannetti G. D. Novembre G. (2023). Spontaneous dyadic behavior predicts the emergence of interpersonal neural synchrony. NeuroImage, 277:120233. 37348621 10.1016/j.neuroimage.2023.120233

[ref32] Kumaravel V. P. Farella E. Parise E. Buiatti M. (2022). NEAR: an artifact removal pipeline for human newborn EEG data. Dev. Cogn. Neurosci. 54:101068. doi: 10.1016/j.dcn.2022.101068, PMID: 35085870 PMC8800139

[ref9009] Lachaux J. P. Rodriguez E. Martinerie J. Varela F. J. (1999). Measuring phase synchrony in brain signals. Hum. Brain Mapp. 8, 194–208. 10619414 10.1002/(SICI)1097-0193(1999)8:4<194::AID-HBM4>3.0.CO;2-C PMC6873296

[ref33] Leong V. Byrne E. Clackson K. Georgieva S. Lam S. Wass S. (2017). Speaker gaze increases information coupling between infant and adult brains. Proc. Natl. Acad. Sci. USA 114, 13290–13295. doi: 10.1073/pnas.1702493114, PMID: 29183980 PMC5740679

[ref34] Leong V. Noreika V. Clackson K. Georgieva S. Brightman L. Nutbrown R. . (2019). Mother-infant interpersonal neural connectivity predicts infants’ social learning.

[ref35] Levy J. Feldman R. (2019). Synchronous interactions Foster empathy. J. Exp. Neurosci. 13:1179069519865799. doi: 10.1177/1179069519865799, PMID: 31384131 PMC6657123

[ref36] Leyton F. Olhaberry M. Alvarado R. Rojas G. Dueñas L. Downing G. . (2019). Video feedback intervention to enhance parental reflective functioning in primary caregivers of inpatient psychiatric children: protocol for a randomized feasibility trial. Trials 20:20. doi: 10.1186/s13063-019-3310-y, PMID: 31088531 PMC6515604

[ref37] Linnér A. Almgren M. (2020). Epigenetic programming-the important first 1000 days. Acta Paediatr. 109, 443–452. doi: 10.1111/apa.15050, PMID: 31603247

[ref38] Lionetti F. Aron A. Aron E. N. Burns G. L. Jagiellowicz J. Pluess M. (2018). Dandelions, tulips and orchids: evidence for the existence of low-sensitive, medium-sensitive and high-sensitive individuals. Transl. Psychiatry 8:24. doi: 10.1038/s41398-017-0090-6, PMID: 29353876 PMC5802697

[ref39] Liu D. Liu S. Liu X. Zhang C. Li A. Jin C. . (2018). Interactive brain activity: review and Progress on EEG-based Hyperscanning in social interactions. Front. Psychol. 9:9. doi: 10.3389/fpsyg.2018.01862, PMID: 30349495 PMC6186988

[ref40] Lordier L. Meskaldji D.-E. Grouiller F. Pittet M. P. Vollenweider A. Vasung L. . (2019). Music in premature infants enhances high-level cognitive brain networks. Proc. Natl. Acad. Sci. USA 116, 12103–12108. doi: 10.1073/pnas.1817536116, PMID: 31138687 PMC6575179

[ref41] Lotzin A. Lu X. Kriston L. Schiborr J. Musal T. Romer G. . (2015). Observational tools for measuring parent-infant interaction: a systematic review. Clin. Child. Fam. Psychol. Rev. 18, 99–132. doi: 10.1007/s10567-015-0180-z, PMID: 25837491

[ref42] Lundqvist P. Weis J. Sivberg B. (2019). Parents’ journey caring for a preterm infant until discharge from hospital-based neonatal home care-a challenging process to cope with. J. Clin. Nurs. 28, 2966–2978. doi: 10.1111/jocn.14891, PMID: 31017322

[ref43] Maitre N. L. Key A. P. Slaughter J. C. Yoder P. J. Neel M. L. Richard C. . (2020). Neonatal multisensory processing in preterm and term infants predicts sensory reactivity and internalizing tendencies in early childhood. Brain Topogr. 33, 586–599. doi: 10.1007/s10548-020-00791-4, PMID: 32785800 PMC7429553

[ref44] Mantis I. Stack D. M. Ng L. Serbin L. A. Schwartzman A. E. (2014). Mutual touch during mother-infant face-to-face still-face interactions: influences of interaction period and infant birth status. Infant Behav. Dev. 37, 258–267. doi: 10.1016/j.infbeh.2014.04.005, PMID: 24793734

[ref45] Miles M. S. Funk S. G. Carlson J. (1993). Parental Stressor Scale: neonatal intensive care unit. Nurs. Res. 42, 148–152. doi: 10.1097/00006199-199305000-00005, PMID: 8506163

[ref46] Montirosso R. Arrigoni F. Casini E. Nordio A. De Carli P. Di Salle F. . (2017). Greater brain response to emotional expressions of their own children in mothers of preterm infants: an fMRI study. J. Perinatol. 37, 716–722. doi: 10.1038/jp.2017.2, PMID: 28151495

[ref47] Montirosso R. Borgatti R. Trojan S. Zanini R. Tronick E. (2010). A comparison of dyadic interactions and coping with still-face in healthy pre-term and full-term infants. Br. J. Dev. Psychol. 28, 347–368. doi: 10.1348/026151009x416429, PMID: 20481392

[ref48] Montirosso R. Rosa E. Giorda R. Fazzi E. Orcesi S. Cavallini A. . (2020). Early parenting intervention – biobehavioral outcomes in infants with neurodevelopmental disabilities (EPI-BOND): study protocol for an Italian multicentre randomised controlled trial. BMJ Open 10:e035249. doi: 10.1136/bmjopen-2019-035249, PMID: 32699128 PMC7375429

[ref49] Mörelius E. Örtenstrand A. Theodorsson E. Frostell A. (2015). A randomised trial of continuous skin-to-skin contact after preterm birth and the effects on salivary cortisol, parental stress, depression, and breastfeeding. Early Hum. Dev. 91, 63–70. doi: 10.1016/j.earlhumdev.2014.12.005, PMID: 25545453

[ref50] Neugebauer C. Oh W. McCarty M. Mastergeorge A. M. (2022). Mother–infant dyadic synchrony in the NICU context. Adv. Neonatal Care 22:170. doi: 10.1097/ANC.0000000000000855, PMID: 35703926

[ref51] Nguyen T. Bánki A. Markova G. Hoehl S. (2020). Studying parent-child interaction with hyperscanning. Prog. Brain Res. 254, 1–24. doi: 10.1016/bs.pbr.2020.05.003, PMID: 32859283

[ref52] Niutanen U. Harra T. Lano A. Metsäranta M. (2020). Systematic review of sensory processing in preterm children reveals abnormal sensory modulation, somatosensory processing and sensory-based motor processing. Acta Paediatr. 109, 45–55. doi: 10.1111/apa.14953, PMID: 31350861

[ref53] Ohuma E. O. Moller A.-B. Bradley E. Chakwera S. Hussain-Alkhateeb L. Lewin A. . (2023). National, regional, and global estimates of preterm birth in 2020, with trends from 2010: a systematic analysis. Lancet 402, 1261–1271. doi: 10.1016/S0140-6736(23)00878-4, PMID: 37805217

[ref54] Pierce S. Kadlaskar G. Edmondson D. A. McNally Keehn R. Dydak U. Keehn B. (2021). Associations between sensory processing and electrophysiological and neurochemical measures in children with ASD: an EEG-MRS study. J. Neurodev. Disord. 13:5. doi: 10.1186/s11689-020-09351-0, PMID: 33407072 PMC7788714

[ref55] Pineda R. Raney M. Smith J. (2019). Supporting and enhancing NICU sensory experiences (SENSE): defining developmentally-appropriate sensory exposures for high-risk infants. Early Hum. Dev. 133, 29–35. doi: 10.1016/j.earlhumdev.2019.04.012, PMID: 31054467

[ref56] Pion-Tonachini L. Kreutz-Delgado K. Makeig S. (2019). ICLabel: an automated electroencephalographic independent component classifier, dataset, and website. NeuroImage 198, 181–197. doi: 10.1016/j.neuroimage.2019.05.026, PMID: 31103785 PMC6592775

[ref57] Pisoni C. Provenzi L. Moncecchi M. Caporali C. Naboni C. Stronati M. . (2021). Early parenting intervention promotes 24-month psychomotor development in preterm children. Acta Paediatr. 110, 101–108. doi: 10.1111/apa.15345, PMID: 32392381

[ref58] Pittet M. P. Vasung L. Huppi P. S. Merlini L. (2019). Newborns and preterm infants at term equivalent age: a semi-quantitative assessment of cerebral maturity. Neuroimage Clin. 24:102014. doi: 10.1016/j.nicl.2019.102014, PMID: 31683202 PMC6838895

[ref59] Porges S. W. Davila M. I. Lewis G. F. Kolacz J. Okonmah-Obazee S. Hane A. A. . (2019). Autonomic regulation of preterm infants is enhanced by family nurture intervention. Dev. Psychobiol. 61, 942–952. doi: 10.1002/dev.21841, PMID: 30868570

[ref60] Poslawsky I. E. Naber F. B. Bakermans-Kranenburg M. J. van Daalen E. van Engeland H. van IJzendoorn M. H. (2015). Video-feedback intervention to promote positive parenting adapted to autism (VIPP-AUTI): a randomized controlled trial. Autism 19, 588–603. doi: 10.1177/1362361314537124, PMID: 24919961

[ref61] Provenzi L. Barello S. Fumagalli M. Graffigna G. Sirgiovanni I. Savarese M. . (2016). A comparison of maternal and paternal experiences of becoming parents of a very preterm infant. J. Obstet. Gynecol. Neonatal. Nurs. 45, 528–541. doi: 10.1016/j.jogn.2016.04.004, PMID: 27266963

[ref62] Provenzi L. Casini E. de Simone P. Reni G. Borgatti R. Montirosso R. (2015). Mother–infant dyadic reparation and individual differences in vagal tone affect 4-month-old infants’ social stress regulation. J. Exp. Child Psychol. 140, 158–170. doi: 10.1016/j.jecp.2015.07.003, PMID: 26247809

[ref63] Provenzi L. Fumagalli M. Bernasconi F. Sirgiovanni I. Morandi F. Borgatti R. . (2017). Very preterm and full-term infants’ response to socio-emotional stress: the role of postnatal maternal bonding. Infancy 22, 695–712. doi: 10.1111/infa.12175, PMID: 33158334

[ref64] Provenzi L. Giorda R. Fumagalli M. Brambilla M. Mosca F. Borgatti R. . (2019). Telomere length and salivary cortisol stress reactivity in very preterm infants. Early Hum. Dev. 129, 1–4. doi: 10.1016/j.earlhumdev.2018.12.002, PMID: 30530269

[ref65] Provenzi L. Giusti L. Caglia M. Rosa E. Mascheroni E. Montirosso R. (2020). Evidence and open questions for the use of video-feedback interventions with parents of children with neurodevelopmental disabilities. Front. Psychol. 11:11. doi: 10.3389/fpsyg.2020.01374, PMID: 32625153 PMC7314919

[ref66] Provenzi L. Giusti L. Fumagalli M. Frigerio S. Morandi F. Borgatti R. . (2019). The dual nature of hypothalamic-pituitary-adrenal axis regulation in dyads of very preterm infants and their mothers. Psychoneuroendocrinology 100, 172–179. doi: 10.1016/j.psyneuen.2018.10.007, PMID: 30343183

[ref67] Provenzi L. Guida E. Montirosso R. (2018). Preterm behavioral epigenetics: a systematic review. Neurosci. Biobehav. Rev. 84, 262–271. doi: 10.1016/j.neubiorev.2017.08.020, PMID: 28867654

[ref68] Provenzi L. Olson K. L. Montirosso R. Tronick E. (2016). Infants, mothers, and dyadic contributions to stability and prediction of social stress response at 6 months. Dev. Psychol. 52, 1–8. doi: 10.1037/dev0000072, PMID: 26569560

[ref69] Provenzi L. Roberti E. Capelli E. (2023). Envisioning translational hyperscanning: how applied neuroscience might improve family-centered care. Soc. Cogn. Affect. Neurosci. 18:nsac061. doi: 10.1093/scan/nsac061, PMID: 36542821 PMC9910277

[ref70] Riva Crugnola C. Ierardi E. Peruta V. Moioli M. Albizzati A. (2021). Video-feedback attachment based intervention aimed at adolescent and young mothers: effectiveness on infant-mother interaction and maternal mind-mindedness. Early Child Dev. Care 191, 475–489. doi: 10.1080/03004430.2019.1652172

[ref71] Santamaria L. Noreika V. Georgieva S. Clackson K. Wass S. Leong V. (2020). Emotional valence modulates the topology of the parent-infant inter-brain network. NeuroImage 207:116341. doi: 10.1016/j.neuroimage.2019.116341, PMID: 31712166

[ref72] Saul M. A. He X. Black S. Charles F. (2022). A two-person neuroscience approach for social anxiety: a paradigm with interbrain synchrony and neurofeedback. Front. Psychol. 12:12. doi: 10.3389/fpsyg.2021.568921, PMID: 35095625 PMC8796854

[ref9010] Spielberger C. D. (1983). State-Trait Anxiety Inventory for Adults (STAI-AD). APA PsycTests.

[ref73] Suir I. Oosterhaven J. Boonzaaijer M. Nuysink J. Jongmans M. (2022). The AIMS home-video method: parental experiences and appraisal for use in neonatal follow-up clinics. BMC Pediatr. 22:338. doi: 10.1186/s12887-022-03398-9, PMID: 35690764 PMC9187888

[ref74] Thomson G. Flacking R. George K. Feeley N. Haslund-Thomsen H. De Coen K. . (2020). Parents’ experiences of emotional closeness to their infants in the neonatal unit: a meta-ethnography. Early Hum. Dev. 149:105155. doi: 10.1016/j.earlhumdev.2020.105155, PMID: 32829240

[ref75] Tronick E. Als H. Adamson L. Wise S. Brazelton T. B. (1978). The infant’s response to entrapment between contradictory messages in face-to-face interaction. J. Am. Acad. Child Psychiatry 17, 1–13. doi: 10.1016/s0002-7138(09)62273-1, PMID: 632477

[ref76] Tryphonopoulos P. D. Letourneau N. (2020). Promising results from a video-feedback interaction guidance intervention for improving maternal-infant interaction quality of depressed mothers: a feasibility pilot study. Can. J. Nurs. Res. 52, 74–87. doi: 10.1177/0844562119892769, PMID: 31910674

[ref9011] Turk E. Endevelt-Shapira Y. Feldman R. van den Heuvel M. I. Levy J. (2022). Brains in sync: Practical guideline for parent–infant EEG during natural interaction. Front. Psychol. 13:833112. 35572249 10.3389/fpsyg.2022.833112 PMC9093685

[ref77] Wass S. V. Noreika V. Georgieva S. Clackson K. Brightman L. Nutbrown R. . (2018). Parental neural responsivity to infants’ visual attention: how mature brains influence immature brains during social interaction. PLoS Biol. 16:e2006328. doi: 10.1371/journal.pbio.2006328, PMID: 30543622 PMC6292577

[ref78] Welch M. G. Ludwig R. J. (2017). Calming cycle theory and the co-regulation of oxytocin. Psychodyn. Psychiatry 45, 519–540. doi: 10.1521/pdps.2017.45.4.519, PMID: 29244620

